# Genomic epidemiology of Colombian resistant *Candida auris* isolates

**DOI:** 10.1128/aac.00725-25

**Published:** 2025-10-07

**Authors:** Maira Lyseth Alvarado Casas, Jeisson Alejandro Triana Díaz, Efrain Montilla-Escudero, Diego Andrés Prada Cardozo, Patricia Escandón Hernández

**Affiliations:** 1 Grupo de Microbiología, Instituto Nacional de Salud 67626, Bogotá, Colombia; University Children's Hospital Münster, Münster, Germany

**Keywords:** *Candida*, epidemiology, antifungal susceptibility, fungal, molecular typing

## Abstract

Since 2016, *Candida auris* has been subject to mandatory reporting in Colombia due to its critical public health implications. In this context, a genomic characterization of clinical *C. auris* isolates collected between 2018 and 2024 through national surveillance led by the Instituto Nacional de Salud (INS) was conducted. Twenty-seven isolates from hospitalized patients across 14 departments were analyzed using next-generation sequencing (NGS) and bioinformatics tools (MycoSNP-nf v1.5, TheiaEuk BLAST, Python script, and MUSCLE, snp_phylogeny) to identify mutations associated with antifungal resistance. The patient group was predominantly male *n* = 18 (66.7%), with 48% (*n* = 13) for the older adult age group (>60 years of age). Antifungal susceptibility testing (anidulafungin, fluconazole, and amphotericin B) revealed that five isolates (18.5%) were resistant to a single antifungal, while 22 (81.5%) were resistant to two. All isolates belonged to clade IV (South America) and exhibited genetic divergence ranging from 5 to 127 single-nucleotide polymorphisms (SNPs). Mutations in the *ERG11* gene were identified in 16 isolates, including Y132F (*n* = 11), G459S (*n* = 1), K143R (*n* = 2), and F444L (*n* = 1), along with a novel mutation, G445D (*n* = 1). Four of these 16 isolates also harbored mutations in the *TAC1B* gene (P595H, *n* = 3; S611P, *n* = 1), and one isolate carried a single *TAC1B* mutation (S195G). Additionally, three isolates presented the S639F mutation in the *FKS1* gene. Notably, seven isolates showed phenotypic resistance without any known resistance mutations. These findings are concerning, as the antifungals involved are critical for treating invasive *Candida* infections. The study highlights the need for ongoing genomic surveillance to complement phenotypic testing and enhance strategies for antifungal resistance control, ultimately informing therapeutic decision-making and drug development.

## INTRODUCTION

*Candida auris* is an emerging fungal pathogen that has generated increasing concern worldwide because of its ability to cause serious nosocomial infections, especially in immunocompromised patients (1, 2). *C. auris* lives on the skin and in the hospital environment, causing frequent outbreaks associated with health care; it is important to note that some clades exhibit resistance to multiple antifungal classes, including azoles (e.g., fluconazole and voriconazole), echinocandins (e.g., anidulafungin and caspofungin), and polyenes (e.g., amphotericin B) (3, 4). The resistance, or multiresistance, of *C. auris* has limited therapeutic options and has worsened infected patients’ clinical progression, posing a critical challenge for health care systems because of the low availability of effective treatments or new therapeutic targets (5). Notably, mutations in the genes responsible for resistance to these antifungals are crucial for understanding the underlying molecular mechanisms and improving treatment strategies.

It is known that antifungal resistance mechanisms can be diverse; however, some of the most common mutations are responsible for an increase in azole resistance genes (*ERG11* and *TAC1B*). The *ERG11* gene encodes lanosterol 14α-demethylase, a key enzyme in the biosynthesis of ergosterol, the main sterol in the fungal cell membrane, in which the mutations modify the enzyme’s structure, altering its affinity for azolic inhibitors, which reduces the effectiveness of these drugs (6, 7). Moreover, *C. auris* has shown resistance to echinocandins, an antifungal class that blocks fungal cell wall synthesis by suppressing β-1,3-glucan synthase, which is encoded by the *FKS1* gene (8). Mutations in *FKS1*, such as S629F and S629Y, have been tied to greater resistance to this class, allowing *C. auris* strains to evade the effects of the treatment and to remain in the hospital environment, which increases the difficulty of reducing infections (9). Resistance to amphotericin B in *C. auris* has been a growing concern, as this drug is considered one of the last lines of defense against serious invasive fungal infections, this resistance is defined by an elevated MIC, meaning that more of the antifungal agent is required to inhibit fungal growth. An isolation is considered resistant to amphotericin B when it has an MIC equal to or greater than 2 µg/mL according to the CDC’s provisional breakpoint which is comparable with EUCAST; however, this resistance can vary among *C. auris* isolates and clades (10). Although the specific resistance mechanisms to amphotericin B in *C. auris* are not completely defined, several have been described, including changes in ergosterol (the main membrane component) that alter the integrity of the plasma membrane, resulting in cell death. Furthermore, mutations in genes encoding a key enzyme in ergosterol synthesis have been described (11).

WGS has been fundamental in the mapping of genetic variations that contribute to antifungal resistance in *C. auris*, providing a more precise understanding of strain diversity and aiding in the prediction of treatment responses. This approach is particularly valuable for epidemiologic surveillance, the monitoring of resistant strains and the design of personalized and targeted therapies (12). Since 2016, the Colombian National Health Institute (INS) has conducted nationwide laboratory-based surveillance of the multidrug-resistant yeast *C. auris*, leading to the identification of approximately 2,500 cases of invasive infection across the country. This surveillance has facilitated epidemiological studies that have identified a geographic substructure with highly related isolates, which supports the hypothesis of local and ongoing transmission (1, 13).

With respect to the molecular mechanisms related to antifungal resistance identified through WGS, one-off gene mutations have been detected. For azole resistance, the most commonly reported mutations include Y132F, F444L, and K143R in the *ERG11* gene, as well as S611P in the *TAC1B* gene. For echinocandin resistance, the S639F(Y) mutation has been identified in the *FSK1* gene. In the case of amphotericin B resistance, mutations have been described in genes encoding transcription factors (*FLO8*) and membrane transport proteins (14–16).

Additionally, molecular docking has emerged as a powerful tool for studying the interactions between antifungals and their molecular targets (16). This computational approach allows modeling of drug binding to key enzymes, such as lanosterol 14α-demethylase and β-1,3-glucan synthase, helping predict how mutations in *ERG11* and *FKS1* may alter these interactions. Mutations occurring in the enzyme’s active site or affecting its formation can modify its affinity for antifungals, reducing their efficacy.

The objective of this study was to conduct a molecular characterization of Colombian *C. auris* isolates to identify mutations associated with antifungal resistance and to evaluate whether these mutations affect the activity of the enzymes and their interaction with the antifungal drug to achieve a better understanding of *C. auris* resistance.

## MATERIALS AND METHODS

### Isolates

A total of 27 *C*. *auris* isolates were selected based on the inclusion criteria: a sequencing algorithm was used that included isolates resistant to one or more antifungal classes. From this population, the selection was random using the conventional *in vitro* method and/or association with the pediatric population. These isolates were obtained through national laboratory-based surveillance led by the Microbiology Group at the Colombian INS between 2018 and 2024, as detailed in Table 1.

**TABLE 1:** Characteristics of *Candida auris* isolates recovered from patients associated with antifungal resistance in Colombia^*a*^

Id	Year	Clade	Department	Country	Sex	Age	Sample type	Outbreak	Antifungal susceptibility MIC value (μg/mL)		
									FCZ	AMB	AND
*B11205*	2013	I	India	–^*b*^	–	–	Chest wound	–	8	0.75	0.5
*B11220*	2008	II	Japan	–	–	–	Auditory canal	–	4	0.38	0.25
*B11227*	2014	III	South Africa	–	–	–	Blood	–	128	0.38	1
*B11245*	2012	IV	Venezuela	–	–	–	Blood	–	–	–	–
*IFRC2087*	2018	V	Iran	–	–	–	Ear discharge	–	–	–	–
Ca05	2018	VI	Singapore	–	–	–	Blood	–	8	2	0.12
*H0059-13-1102*	2018	IV	Bogotá	Colombia	Male	51	Orotracheal secretions	No	32	>2	>0.125
*H0059-13-1162*	2019	IV	Bolívar, Cartagena	Colombia	Male	2	Urine from catheter	No	>2.0	>2	>2.0
*H0059-13-1163*	2019	IV	Bolívar, Cartagena	Colombia	Male	2	Urine from catheter	No	>4.0	>2	>2.0
*H0059-13-1171*	2019	IV	Huila, Neiva	Colombia	Male	67	Blood	No	32	>2	>0.125
*H0059-13-1203*	2019	IV	Antioquia, Medellín	Colombia	Female	81	Underarm swabs	No	4	2	0.25
*H0059-13-2243*	2021	IV	Cesar, Valledupar	Colombia	Female	68	Blood	No	>64	>16	0.125
*H0059-13-2249*	2021	IV	Santander, Bucaramanga	Colombia	Male	0.11	Blood	No	>64	>16	0.125
*H0059-13-2265*	2021	IV	Bogotá	Colombia	Male	45	Urine from catheter	No	>64	2	1
*H0059-13-2283*	2021	IV	Bolívar, Cartagena	Colombia	Male	77	Urine from catheter	No	1	4	16
*H0059-13-2337*	2021	IV	Cesar, Valledupar	Colombia	Female	42	Blood	No	>64	2	0.5
*H0059-13-2609*	2021	IV	Tolima, Ibagué	Colombia	Male	29	Groin swabs	si	>64	2	0.5
*H0059-13-2672*	2021	IV	Norte de Santander, Cúcuta	Colombia	Male	1	Blood	si	32	1	4
*H0059-13-2756*	2022	IV	Norte de Santander, Cúcuta	Colombia	Male	29	Urine from catheter	No	32	2*	0.5
*H0059-13-2815*	2022	IV	Boyacá, Tunja	Colombia	Male	76	Blood	si	>64	4	0.25
*H0059-13-2930*	2022	IV	Atlántico, Barranquilla	Colombia	Male	73	Urine from catheter	No	>64	>16	0.25
*H0059-13-2971*	2022	IV	Atlántico, Barranquilla	Colombia	Male	42	Blood	si	>64	16	0.25
*H0059-13-2989*	2022	IV	Bogotá	Colombia	Female	< 0.01	Blood	No	>64	4	0.25
*GMR-OM-209-23*	2023	IV	Tolima, Ibagué	Colombia	Female	64	Blood	No	>64	8	0.25
*GMR-OM-210-23*	2023	IV	Tolima, Ibagué	Colombia	Male	93	Urine from catheter	No	>64	8	0.25
*GMR-OM-275-23*	2023	IV	Quindío, Armenia	Colombia	Male	79	Blood	si	32	2	0.125
*GMR-OM-639-23*	2023	IV	Córdoba, Montería	Colombia	Male	3	Blood	No	256	2	0.25
*GMR-OM-976-23*	2023	IV	Atlántico, Barranquilla	Colombia	Female	49	Urine from catheter	No	256	2	0.25
*GMR-OM-896-23*	2023	IV	Valle del Cauca, Cali	Colombia	Male	1	Blood	si	>64	4	0.5
*GMR-OM-576-24*	2024	IV	Bolívar, Cartagena	Colombia	Female	70	Blood	No	>64	4	0.25
*GMR-OM-933-24*	2024	IV	Meta, Villavicencio	Colombia	Male	82	Blood	No	>64	4	1
*GMR-OM-024-24*	2024	IV	Bolívar, Cartagena	Colombia	Female	60	Blood	No	4	0.5	4
*GMR-OM-065-24*	2024	IV	Bolívar, Cartagena	Colombia	Female	73	Blood	No	1	1	8

^*a*^ Minimum Inhibitory Concentration (MIC); fluconazole (FCZ); amphotericin B (AMB); anidulafungin (AND); indicates resistance.

^*b*^ –, no information available.

The minimum inhibitory concentration (MIC) was determined for three antifungal classes: echinocandins (anidulafungin), azoles (fluconazole), and polyenes (amphotericin B). MICs were assessed using the broth microdilution method, following the M27-A3 guidelines of the Clinical and Laboratory Standards Institute (CLSI) (17), or via the colorimetric method with Sensititre YeastOneYO9 susceptibility plates (TREK Diagnostics Systems, West Sussex, UK). The tentative breakpoints used for interpretation were those established by CDC (10). Isolates were classified as resistant if they presented an MIC ≥4 µg/mL for anidulafungin, a MIC ≥2 µg/mL for amphotericin B, and a MIC ≥32 µg/mL for fluconazole.

### DNA extraction and WGS

Genomic DNA was extracted via a Quick_DNA Fungal/Bacterial Microprep Kit (Zymo Research, Irvine, CA, USA) following the manufacturer’s instructions with minor modifications. Genomic libraries were constructed using Nextera XT Paired-end (Illumina, San Diego, CA, USA) and sequenced on a MiSeq platform (2 × 250 bp) at 80× coverage. All the isolates exhibited high-quality sequencing metrics. Additionally, reference genomes from the five previously described *C. auris* clades were included: clade I (India, B11205), clade II (Japan, B11220), clade III (South Africa, B11227), clade IV (Venezuela, B11245), clade V (Iran, IFC2087), and clade VI (Singapore, Ca05) (18, 19).

### Phylogenetic analysis

The phylogenetic analysis was conducted using the maximum likelihood method in IQ-TREE software, which is based on single nucleotide polymorphisms (SNPs). To remove positions that had <10× coverage, <90% variant allele calls, or that were identified by Nucmer as being within duplicated regions in the reference, the reference genome used for the analyses was B11245 (South American clade IV) with GenBank Accession GCA_008275145.1. The analysis followed the SNP phylogeny workflow (https://gitlab.com/cgps/ghru/pipelines/snp_phylogeny), and the results were visualized using the Microreact application (20).

### Single-nucleotide polymorphism detection using MycoSNP

In addition to the 27 *C*. *auris* isolates sequenced in this study, the reference genome B11245 (GCA_008275145.1) from clade IV (Venezuela) was used for read mapping and SNP calling. The analysis was performed using the MycoSNP-nf v1.5 workflow.

Additionally, TheiaEuk workflow data were analyzed using the Terra platform to identify mutations associated with antifungal resistance via alignment and calling of variants in the reference genes *ERG11* (lanosterol 14α-demethylase), *FKS1* (1,3-b-glucan synthase), and *FUR1* (uracil phosphoribosyltransferase). Additionally, other *C. auris* genes associated with antifungal resistance, such as *ERG2* and *ERG3* associated with loss-of-function mutations, *ERG5* mutations leading to steroid precursor accumulation, *ERG6* mutations altering ergosterol structure, *FLO8* mutations contributing to biofilm formation and persistence in the hospital environment, and *TAC1a* and *TAC1b* mutations in zinc cluster transcription factor genes, were analyzed. Gene identification and mapping were performed using BLAST. In this process, tblastn was used to compare protein query sequences against a reference database containing nucleotide sequences.

The alignment coordinates for each identified gene in the database were extracted from the BLAST results. The alignments were filtered such that only those with an identity and coverage percentage of ≥90% were retained. Next, a Python script utilizing the Biopython library was employed to retrieve the DNA sequences corresponding to the mapped regions. These sequences were then translated into proteins, following the appropriate reading frame. Finally, multiple alignment of the obtained protein sequences was performed using MUSCLE to analyze conservation and possible conservation.

### Docking analysis

#### Identification of the *ERG11* and *FKS1* active sites

The active sites of the molecules lanosterol 14α-demethylase (*ERG11*) and 1,3-b-glucan synthase (*FKS1*) were identified from their three-dimensional structures via the COACH server (meta-server approach to protein‒ligand binding site prediction). This analysis identified the active sites or binding sites of the proteins, which allowed an analysis of the docking targeted toward these residues.

### Molecular docking

The server HADDOCK 2.4 (High Ambiguity Driven protein‒protein DOCKing https://rascar.science.uu.nl/haddock2.4/) was used to evaluate the molecular docking between the antifungals (fluconazole and anidulafungin) and the proteins lanosterol 14α-demethylase (*ERG11*) and 1,3-b-glucan synthase (*FKS1*). To optimize HADDOCK entry parameters, the analysis option “Protein or Protein-Ligand” was selected, the three-dimensional structures of the molecules lanosterol 14α-demethylase (*ERG11*) and 1,3-b-glucan synthase (*FKS1*) were selected as macromolecules (Protein), and the three-dimensional structures of the antifungals (fluconazole and anidulafungin) were selected as ligands (Protein-Ligand). The amino acid residues related to the active sites of lanosterol 14α-demethylase (*ERG11*) and 1,3-b-glucan synthase (*FKS1*) were selected for analysis, which allowed docking targeting the binding sites. Equally, as an analysis control, a blind docking experiment was performed, in other words, using all the 14α-demethylase (*ERG11*) and 1,3-b-glucan synthase (*FKS1*) amino acid residues, with the objective of determining whether the antifungals (fluconazole and anidulafungin) bind to a region different from the active sites. Finally, the interactions between the molecules 14α-demethylase (*ERG11*) and 1,3-b-glucan synthase (*FKS1*), the antifungals (fluconazole and anidulafungin) and the mutations found in this study were visualized via PyMOL software (https://www.pymol.org).

## RESULTS

### Isolate description

*C. auris* isolates were obtained from patients hospitalized in medical centers located in 14 departments of Colombia, as shown in Table 1.

Among all the patients, 66.7% (*n* = 18) were male, and 33.3% (*n* = 9) were female. With respect to age distribution, 26% (*n* = 7) were in early childhood (≤1 month–3 years of age), 26% (*n* = 7) were in adulthood (29–51 years of age), and 48%(*n* = 13) were elderly (60–93 years of age). The majority of the isolates were derived from samples such as blood cultures 59.3% (*n* = 16) (Table 1).

### Antifungal susceptibility

Among the isolates, five (18.5%) were resistant to a single class of antifungal agent: two were resistant to anidulafungin, with minimum inhibitory concentrations (MICs) of 4 and 8 µg/mL, whereas three were resistant to amphotericin B, with MICs greater than 2 µg/mL. On the other hand, 22 (81.5%) isolates were resistant to two classes of antifungals: 1 to amphotericin B and anidulafungin (MICs of 4 µg/mL and 16 µg/mL, respectively), 1 to fluconazole and anidulafungin (MICs of 32 µg/mL and 4 µg/mL, respectively), and 20 to fluconazole and amphotericin B (MICs of 32–256 µg/mL and 2–16 µg/mL, respectively).

### Whole-genome sequencing

**Fig 1:**
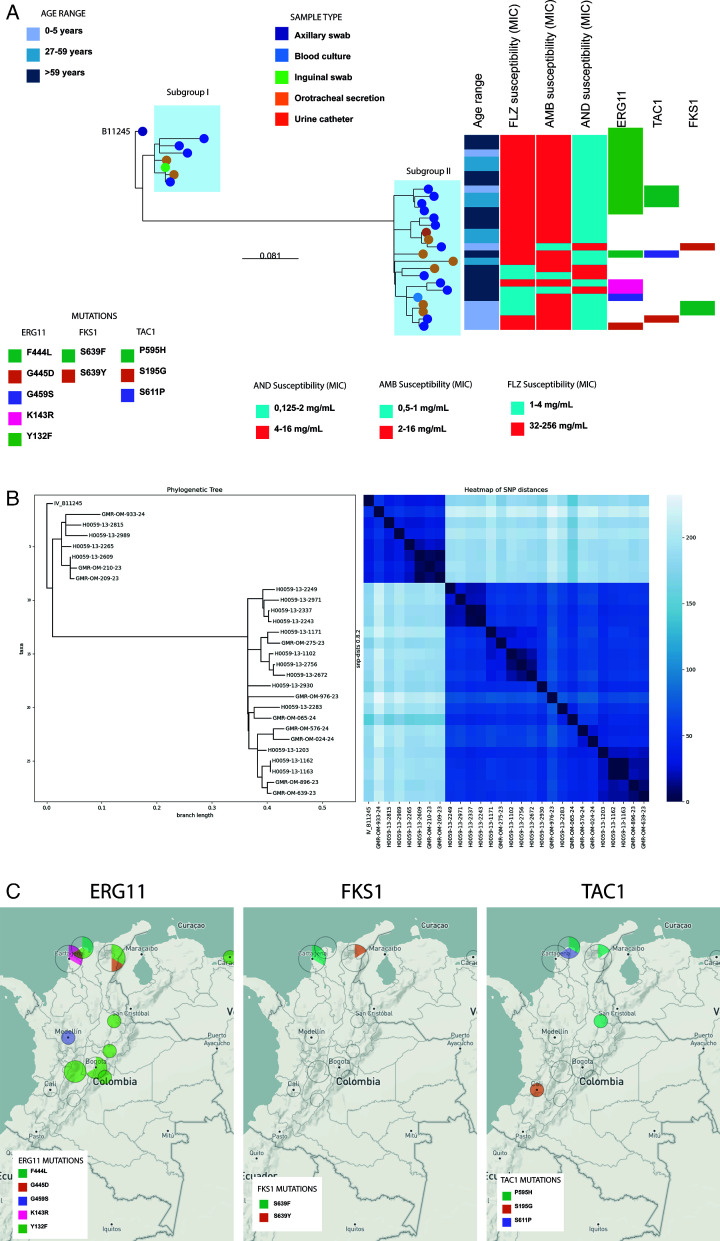
Phylogenetic tree of *C. auris* isolates. (**A**) Distances between reference B11245, clade IV and Colombian isolates. (**B**) Heatmap of distances of SNPs in clade IV between all *C. auris* isolates studied. (**C**) Geographic distribution of mutations in isolates resistant to each antifungal drug.

Phylogenetic analysis revealed that the 27 *C*. *auris* genomes clustered within clade IV. Additionally, a clear phylogeographic structure was observed, comprising two distinct subgroups separated by approximately 79 SNPs, representing different geographical regions. Subgroup I corresponded to the central region of Colombia, encompassing the departments of Boyacá, Bogotá, Tolima, and Meta, with seven isolates, whereas subgroup II included the departments of Atlántico, Cesar, Córdoba, Bolivar, Norte de Santander, Antioquia, Santander, Bogotá, Quindío, Valle del Cauca, and Huila, with 20 isolates. Notably, the Bogotá isolate in subgroup II was the only one obtained from an orotracheal secretion sample. Within each subgroup, significantly fewer SNPs were identified. In subgroup I, the SNP range varied between 4 and 56, where ≤ 11 SNPs differentiated three isolates from Tolima. In subgroup II, the SNP range was from 5 to 79, where ≤16 SNPs distinguished 8 isolates from Cesar, Bogotá, Norte de Santander, Córdoba, and Valle del Cauca (Fig. 1).

Sequencing and analysis of genes involved in antifungal resistance revealed the presence of previously reported mutations as well as novel single-nucleotide polymorphisms (SNPs). In the *ERG11* gene, which is associated with azole resistance, 16 (59.2%) *C*. *auris* isolates that presented unique mutations in this gene (Y132F, F444L, K143R, and G445D) were identified. Among these isolates, 14 were resistant to azoles, with MICs greater than 64 µg/mL and up to 256 µg/mL (Table 2). The two fluconazole-sensitive isolates presented mutations such as K143R and G459S in the *ERG11* gene.

**TABLE 2:** *Candida auris* isolates from Colombia with mutations associated with azoles

ID	Department	Year	Sample type	Antifungal susceptibility MIC^*a*^			Mutation ERG11
				FCZ^*b*^ (µg/mL)	AMB^*c*^ (µg/mL)	AND^*d*^ (µg/mL)	
*B11243*	Venezuela	2013	Blood	ND^*g*^	ND	ND	Y132F
*H0059-13-1203*	Antioquia	2019	Underarm swabs	4	2^*e*^	0.25	G459S
*H0059-13-2265*	Bogotá	2021	Urine from catheter	>64^*e*^	2^*e*^	1	Y132F
*H0059-13-2989*		2022	Blood	>64^*e*^	4^*e*^	0.25	Y132F
*H0059-13-2243*	Cesar	2021	Blood	>64^*e*^	>16^*e*^	0.125	Y132F
*H0059-13-2337*		2021	Blood	>64^*e*^	2^*e*^	0.5	Y132F
*H0059-13-2249*	Santander	2021	Blood	>64^*e*^	>16^*e*^	0.125	Y132F
*H0059-13-2609*	Tolima	2021	Groin swabs	>64^*e*^	2^*e*^	0.5	Y132F
*GMR-OM-209-23*		2023	Blood	>64^*e*^	8^*e*^	0.25	Y132F
*GMR-OM-210-23*		2023	Urine from catheter	>64^*e*^	8^*e*^	0.25	Y132F
*H0059-13-2971*	Atlántico	2022	Blood	>64^*e*^	16^*e*^	0.25	Y132F
*H0059-13-2930*		2022	Urine from catheter	>64^*e*^	16^*e*^	0.25	F444L
*H0059-13-2815*	Boyacá	2022	Blood	>64^*e*^	4^*e*^	0.25	Y132F
*GMR-OM-639-23*	Córdoba	2023	Blood	256^*e*^	2^*e*^	0.25	**G445D^*f*^**
*GMR-OM-933-24*	Meta	2024	Blood	>64^*e*^	4^*e*^	1	Y132F
*GMR-OM-024-24*	Bolívar	2024	Blood	4	0.5	4	K143R
*GMR-OM-576-24*	Bolívar	2024	Blood	>64^*e*^	4^*e*^	0.25	K143R

^*a*^ Minimal inhibitory concentration (MIC).

^*b*^ Fluconazole (FCZ).

^*c*^ Amphotericin B (AMB).

^*d*^ Anidulafungin (AND).

^*e*^ Indicates resistance.

^*f*^ Bold indicates new mutation found in Colombian isolate.

^*g*^ ND, no data.

With respect to echinocandin resistance, three (11.1%) isolates were found to have mutations in the *FKS1* gene (S639F, S639Y), of which only one was resistant to anidulafungin, with an MIC of 4 µg/mL (Table 3). Moreover, eight *C. auris* isolates did not have mutations but were resistant on the basis of susceptibility testing: one was resistant to anidulafungin, with an MIC of 8 µg/mL, and six were multidrug resistant, showing resistance to fluconazole and amphotericin B, with MICs of 32–256 µg/mL and 2–16 µg/mL, respectively. One additional isolate was resistant to amphotericin B and anidulafungin, with MICs of 4 µg/mL and 16 µg/mL, respectively.

**TABLE 3:** *Candida auris* isolates from Colombia with mutations associated with echinocandins

ID	Department	Year	Sample type	Antifungal susceptibility MIC^*a*^			Mutation FKS1
				FCZ^*b*^ (µg/mL)	AMB^*c*^ (µg/mL)	AND^*d*^ (µg/mL)	
*H0059-13-1162*	Bolívar	2019	Urine from catheter	>2	>2^*e*^	>2	S639F
*H0059-13-1163*	Bolívar	2019	Urine from catheter	>4	>2^*e*^	>2	S639F
*H0059-13-2672*	Norte de Santander	2021	Blood	32^*e*^	1	4^*e*^	S639Y

^*a*^ Minimal inhibitory concentration (MIC).

^*b*^ Fluconazole (FCZ).

^*c*^ Amphotericin B (AMB).

^*d*^ Anidulafungin (AND).

^*e*^ Indicates resistance.

Notably, this study identified an amino acid substitution in a key region (Hot Spot 3) of the *ERG11* gene (G445D), which may be associated with fluconazole resistance in *C. auris*. This substitution was found in an isolate from the department of Cordoba that was recovered from a pediatric patient.

We found that 21 (77.8%) of the 27 isolates resistant to fluconazole had known mutations in the *ERG11* and/or *TAC1b* genes, whereas no mutations were detected in the remaining isolates. Additionally, two isolates that were susceptible to fluconazole *in vitro* presented mutations in the *ERG11* gene.

All the isolates from subgroup I carried only the *Y132F* mutation; in contrast, those from subgroup II presented greater diversity, with variations identified in the *ERG11*, *TAC1B*, and *FKS1* genes.

Alignment and analysis of the various genes revealed that, compared with the reference sequence B11245, our 27 isolates shared 98%–100% identity. In *ERG2*, *ERG3*, *ERG5*, *ERG6*, *TAC1a*, and *FLO8*, no mutations were detected in critical regions associated with antifungal resistance to azoles or polyenes. This was particularly notable for the *FLO8* gene despite most isolates being resistant to amphotericin B. However, for the *ERG5* gene, a series of mutations were observed in the initial region of the protein, which were common among some isolates, as shown in Table 4.

**TABLE 4:** *Candida auris* isolates from Colombia with mutations in the start region of the protein of the ERG5 gene

ID	Department	Year	Sample type	Antifungal susceptibility MIC^*a*^			Mutation ERG5
				FCZ^*b*^ (µg/mL)	AMB^*c*^ (µg/mL)	AND^*d*^ (µg/mL)	
*H0059-13-2243*	Cesar	2021	Blood	>64^*e*^	>16^*e*^	0.125	P5T, T6D, E7R, K8E, A9S
*H0059-13-2265*	Bogotá	2021	Urine from catheter	>64^*e*^	2^*e*^	1	
*H0059-13-2815*	Boyacá	2022	Blood	>64^*e*^	4^*e*^	0.25	
*H0059-13-2971*	Atlántico	2022	Blood	>64^*e*^	16^*e*^	0.25	
*GMR-OM-209-23*	Tolima	2023	Blood	>64^*e*^	8^*e*^	0.25	
*GMR-OM-576-24*	Bolívar	2024	Blood	>64^*e*^	4^*e*^	0.25	E7R y A9Q

^*a*^ Minimal inhibitory concentration (MIC).

^*b*^ Fluconazole (FCZ).

^*c*^ Amphotericin B (AMB).

^*d*^ Anidulafungin (AND).

^*e*^ Indicates resistance.

With respect to the *TAC1b* gene, three mutations were observed: those that have already been reported in other studies (S195G, S611P, and P595H) and are present in five different isolates from four departments (Table 5).

**TABLE 5:** *Candida auris* isolates from Colombia with mutations in TAC1b

ID	Department	Year	Sample type	Antifungal susceptibility MIC^*a*^			Mutation TAC1b
				FCZ^*b*^ (µg/mL)	AMB^*c*^ (µg/mL)	AND^*d*^ (µg/mL)	
*H0059-13-2249*	Santander	2021	Blood	>64^*e*^	>16^*e*^	0.125	P595H
*H0059-13-2337*	Cesar	2021	Blood	>64^*e*^	2^*e*^	0.5	P595H
*H0059-13-2930*	Atlántico	2022	Urine from catheter	>64^*e*^	16^*e*^	0.25	S611P
*H0059-13-2971*	Atlántico	2022	Blood	>64^*e*^	16^*e*^	0.25	P595H
*GMR-OM-896-23*	Valle del Cauca	2023	Blood	>64^*e*^	4^*e*^	0.5	S195G

^*a*^ Minimum concentration inhibition (MIC).

^*b*^ Fluconazole (FCZ).

^*c*^ Amphotericin B (AMB).

^*d*^ Anidulafungin (AND).

^*e*^ Indicates resistance.

### Molecular docking analysis

Molecular docking was performed for two antifungal compounds (fluconazole and anidulafungin) with the proteins lanosterol 14α-demethylase (*ERG11*) and β-1,3-glucan synthase (*FKS1*). Ligand affinity energies were calculated for these proteins: for 14α-demethylase (*ERG11*) with the fluconazole molecule, the binding energy was −7 kcal/mol, indicating a relatively strong favorable interaction. For β-1,3-glucan synthase (*FKS1*) with the anidulafungin molecule, the binding energy was −9.2 kcal/mol, reflecting a very strong interaction.

**Fig 2:**
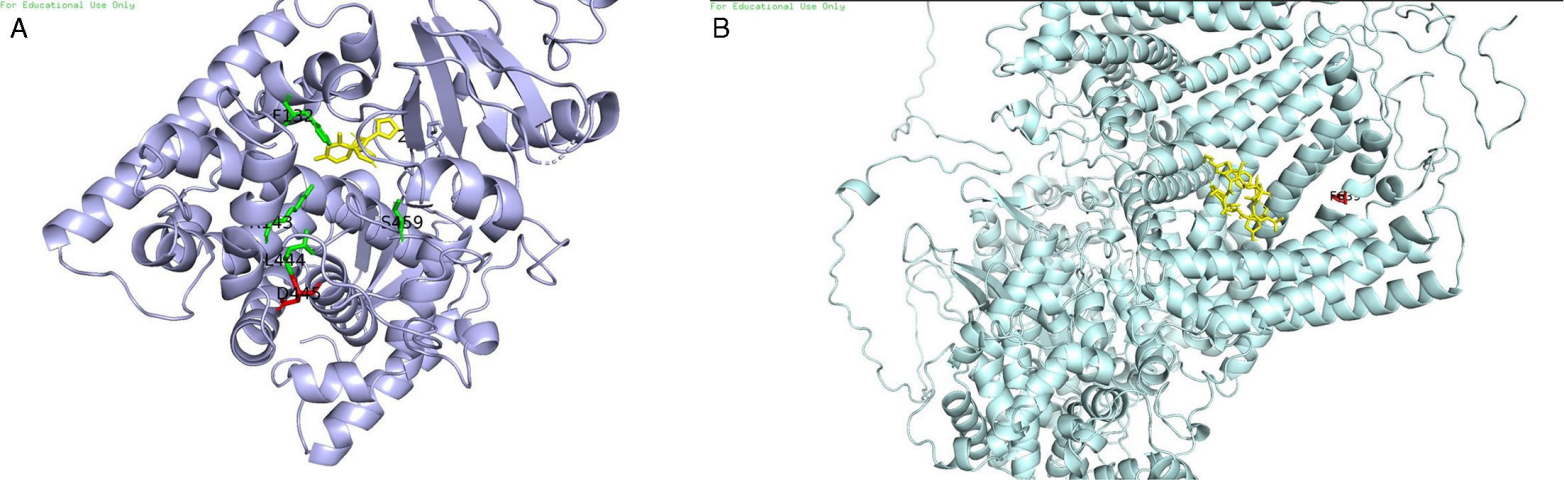
Molecular docking analysis visualization of the (**A**) lanosterol 14α-demethylase (*ERG11*) enzyme with the mutations found in this study. The mutations reported in the literature are in green (Y132F, G459S, F444L, and K143R). The novel mutation (G445D) is shown in red, and the antifungal drug fluconazole is shown in yellow. (**B**) β-1,3-Glucan synthase (*FKS1*) enzyme with the mutations found in this study. Those mutations previously described in the literature are in red (S639F, S639Y), and the antifungal drug anidulafungin is shown in yellow.

When the effects of substitutions on protein function were analyzed, it was found that among the five *ERG11* substitutions, Y132F, F444L, and G445D were the ones most likely to affect enzyme activity due to changes in the active site, potentially altering flexibility and electrostatic interactions. For *FKS1*, the two observed substitutions affected protein activity, as they were located in a critical functional region (Fig. 2).

## DISCUSSION

The isolates obtained from hospitalized patients in 14 departments in Colombia revealed an important incidence of *C. auris* infections in different regions of the country, with a predominant distribution in males (66.7%) and a high percentage of adults (48%). This pattern is consistent with the findings of previous studies suggesting that *Candida* infections have a greater impact on the older population, who have a compromised immune system (21, 22). The equal distribution between early childhood and adulthood (26%) also highlights the relevance of *C. auris* as a nosocomial pathogen that affects vulnerable groups as well as the general population in Colombia (1, 5, 23). Moreover, the wide geographic distribution of the isolates highlights the importance of monitoring and controlling the spread of this species, which stands out for its ability to survive in hospital settings (1, 24).

The antifungal resistance profile reported in this study is alarming, with a high percentage of isolates being resistant to multiple antifungal classes. In particular, resistance to fluconazole, amphotericin B, and anidulafungin, which affects more than 80% of the isolates, is a growing threat that *C. auris* represents to public health. This finding is in line with that reported by Escandón and collaborators in the surveillance of *Candida auris* in Colombia in 2021, where high MICs were observed for these antifungals, and 1 in 10 isolates tested showed multiresistance to two antifungals (1); similarly, other authors reported the same multiresistance behavior in *C. auris* isolates (22, 23, 25). Although the introduction of other *C. auris* clades has not been observed in Colombia, monitoring and awareness of the indiscriminate use of the drugs available for the treatment of these infections are important, as this is one of the main causes of the increase in resistance observed today.

Early diagnosis of *Candida* infections is crucial since a delay in initiating antifungal treatment is associated with a significant increase in mortality (26). While the results of microbiological studies and, in some cases, molecular tests are obtained, it is necessary to implement empirical antifungal treatment guided by clinical presentation and candidemia prediction indices (26). In Colombia, the use of fluconazole and amphotericin B as antifungal agents continues to be frequent despite growing concern about resistance. Susceptibility to fluconazole varies significantly between regions and *Candida* species: studies in Bogotá report resistance of up to 30% and in Medellín 22%, especially among non-albicans species (27, 28). Nationally, fluconazole remains the most prescribed antifungal, even for *C. auris* infections, as evidenced by a multicenter study in high-complexity hospitals where approximately 43% of patients received fluconazole at the time of a positive blood culture (29). Despite international recommendations prioritizing the use of echinocandins against multidrug-resistant species, their use remains limited in Colombia, possibly due to economic and availability barriers (30).

In contrast, amphotericin B, although associated with greater toxicity, remains a viable alternative in scenarios of resistance or lack of access to echinocandins (26).

The WGS results also provide some key information about the genetic and phylogeographic diversity of *C. auris* isolates in Colombia. The distinction of the isolates into two subgroups within clade IV (subgroup I and subgroup II) clearly reveals geographic differences that indicate possible localized transmission sources. This finding is in accordance with previous studies that revealed the existence of different *C. auris* lineages in various parts of the world. This suggests that the spread of the pathogen could be influenced by local epidemiologic and genetic factors (7, 31). Additionally, the *C. auris* strains analyzed in this study with amphotericin B and fluconazole resistance were isolated from different regions of Colombia, which is different from the findings of previous studies, where the *C. auris* strains were mainly isolated from the north coast and central region of the country (1, 14, 21, 22)

Among the five substitutions observed in the *ERG11* gene, the most frequent one was Y132F, which is particularly relevant because it is found in the enzyme’s active site. This mutation induces structural changes in *ERG11*, altering its interaction with fluconazole, which has been widely reported in *Candida* species with antifungal resistance; this finding corroborates the high MIC values observed in the susceptibility tests in this study (32). These findings reinforce the idea that mutations in *ERG11* are key factors in azole resistance in *C. auris*. Interestingly, the substitution G445D was found in only one isolate collected from a minor in the department of Córdoba, which indicates possible regional differences in resistance patterns that could be related to the local evolution of the pathogen (33). Genetic analysis using multiple alignment of the target gene sequences revealed that the amino acid lengths between the reference sequences and our isolates, as well as the reference for clade IV (B11245), were >90%, which means that they are highly similar.

Molecular analysis of the mutations in genes related to antifungal resistance, such as *ERG5* and *TAC1b*, revealed that some of these mutations are common in the isolates, which suggests that certain resistance mechanisms could be shared between different local *C. auris* strains in Colombia. These mutations have previously been associated with changes in ergosterol synthesis and biofilm formation, both of which are key factors in pathogenicity and resistance to antifungal treatment (15, 25). Additionally, the identification of mutations in critical regions of the proteins *ERG11* and *FKS1*, which affect enzymatic activity and molecular interactions, highlights the importance of these genes as potential targets for the design of new antifungal therapies (4, 6, 8). Most of the isolates with mutations in *ERG11* (Y132F) and *TAC1b* (P595H, S611P, S195G) presented elevated MICs for fluconazole and amphotericin B, which suggests that new mutation combinations could contribute to the observed increase in MICs in the last few years. These findings support the hypothesis that the appearance of new resistance mechanisms could explain the increasing resistance of *C. auris* to different antifungal classes (1, 31, 34). Additionally, the second most common genotype found in the Colombian isolates resistant to fluconazole was *ERG11* (F444L) and *TAC1b* (S611P), even though it was observed in only one isolate, which suggests a lower prevalence in comparison with other mutations. The identification of these mutations in *C. auris* in Colombia reinforces the need for continuous surveillance and the implementation of stricter control strategies in hospitals.

Although the mechanisms responsible for resistance to polyenes in *C. auris* are not yet understood, recent studies have identified mutations that lead to the overexpression of various genes (*ERG2*, *ERG3*, *ERG5*, *ERG11*) and transcription factors, such as *FLO8*, which are involved in the stability of the cellular membrane in Candida species (35). In this study, however, mutations in *ERG11* and *ERG5* were detected, which likely contributed to the reduction in susceptibility to fluconazole and amphotericin B, possibly as a result of the decrease in ergosterol in the cell membrane of these isolates (36).

Some isolates had an indel-type mutation, followed by substitutions in the *ERG5* gene. Although these mutations have not been reported previously, they may have contributed to the amphotericin B resistance observed in the phenotypic tests, as *ERG5* performs key functions in ergosterol metabolism. Therefore, it is essential to perform gene expression studies to confirm this hypothesis and to better understand the molecular mechanisms involved. These results highlight the need to continue researching other possible resistance mechanisms in *C. auris*. In future studies, it would be important in future studies to be able to analyze and evaluate the composition of the sterols that are part of the cell membrane of strains with different susceptibility profiles and specific genetic variations, such as SNPs, to demonstrate whether there is a limitation, which would allow a more precise diagnosis of resistance to amphotericin (37).

With respect to echinocandin resistance, mutations in *FKS1*, particularly at position S639, were the most observed mutations in the *C. auris* isolates studied. These mutations occur in the Hot Spot 1 region (HS1) in *FKS1*, an important region for β-1,3-glucan synthase activity, altering the structure of the enzyme and limiting its affinity for the echinocandins. Additionally, these mutations can predispose the cell to some azoles, as observed in this study (32). The information derived from the docking analysis also provides valuable insight into how mutations could affect the efficacy of antifungals, which could open new paths for the development of more effective treatments (16).

It is important in future studies to evaluate the conformational variability between the studied isolates of our clade IV, to correlate this variability with the resistant phenotype through mutations in proteins of great clinical importance such as *ERG11* and *FKS1* that affect the antifungal susceptibility available for the treatment of infections caused by *Candida*. Studies have already reported that mutations such as Y132F and K143R, also found in this study, cause high variability in these target regions and, therefore, affect the interactions between the drug and the enzyme; this occurs due to the alteration of the hydrogen bond network mediated by water in short-tailed triazoles such as fluconazole, which would be of great relevance to know for the new mutation found for the *ERG11* gene (G445D). Regarding resistance to echinocandins, it has been reported that the S639F, S639Y mutations found in Colombian isolates in this study make these variable regions more rigid and stabilize the α-helix type conformations and it is suggested that the conformational variability of HS1 is one of the factors that influence the Eagle effect with the dispersion of the MICs (38).

In conclusion, the findings of this study help elucidate the epidemiology, resistance, and molecular genetics of *C. auris* in Colombia, as well as the molecular mechanisms involved in its antifungal resistance. Owing to the growing threat of resistant infections, the incorporation of methodologies such as genomic sequencing is crucial for the development of new treatments and for controlling the spread of *C. auris*.

## Data Availability

The sequencing data have been deposited in the Sequence Read Archive (SRA) of the National Center for Biotechnology Information (NCBI) under BioProject PRJNA846619.

## References

[1] Escandón P, Cáceres DH, Lizarazo D, Lockhart SR, Lyman M, Duarte C. 2022. Laboratory-based surveillance of Candida auris in Colombia, 2016-2020. Mycoses 65:222–225. doi:10.1111/myc.13390 34731508 PMC9299663

[2] Cristina ML, Spagnolo AM, Sartini M, Carbone A, Oliva M, Schinca E, Boni S, Pontali E. 2023. An overview on Candida auris in healthcare settings. J Fungi 9:913. doi:10.3390/jof9090913 PMC10532978 37755021

[3] Lockhart SR. 2019. Candida auris and multidrug resistance: defining the new normal. Fungal Genet Biol 131:103243. doi:10.1016/j.fgb.2019.103243 31228646 PMC12012538

[4] Lockhart SR, Etienne KA, Vallabhaneni S, Farooqi J, Chowdhary A, Govender NP, Colombo AL, Calvo B, Cuomo CA, Desjardins CA, Berkow EL, Castanheira M, Magobo RE, Jabeen K, Asghar RJ, Meis JF, Jackson B, Chiller T, Litvintseva AP. 2017. Simultaneous emergence of multidrug-resistant Candida auris on 3 continents confirmed by whole-genome sequencing and epidemiological analyses. Clin Infect Dis 64:134–140. doi:10.1093/cid/ciw691 27988485 PMC5215215

[5] Escandón P, Lockhart SR, Chow NA, Chiller TM. 2023. Candida auris: a global pathogen that has taken root in Colombia. Biomedica 43:278–287. doi:10.7705/biomedica.7082 37721898 PMC10599714

[6] Chowdhary A, Prakash A, Sharma C, Kordalewska M, Kumar A, Sarma S, Tarai B, Singh A, Upadhyaya G, Upadhyay S, Yadav P, Singh PK, Khillan V, Sachdeva N, Perlin DS, Meis JF. 2018. A multicentre study of antifungal susceptibility patterns among 350 Candida auris isolates (2009-17) in India: role of the ERG11 and FKS1 genes in azole and echinocandin resistance. J Antimicrob Chemother 73:891–899. doi:10.1093/jac/dkx480 29325167

[7] Rybak JM, Muñoz JF, Barker KS, Parker JE, Esquivel BD, Berkow EL, Lockhart SR, Gade L, Palmer GE, White TC, Kelly SL, Cuomo CA, Rogers PD. 2020. Mutations in TAC1B: a novel genetic determinant of clinical fluconazole resistance in Candida auris. mBio 11:e00365-20. doi:10.1128/mBio.00365-20 32398311 PMC7218281

[8] Kordalewska M, Lee A, Park S, Berrio I, Chowdhary A, Zhao Y, Perlin DS. 2018. Understanding echinocandin resistance in the emerging pathogen Candida auris. Antimicrob Agents Chemother 62:e00238-18. doi:10.1128/AAC.00238-18 29632013 PMC5971591

[9] Chow NA, Muñoz JF, Gade L, Berkow EL, Li X, Welsh RM, Forsberg K, Lockhart SR, Adam R, Alanio A, Alastruey-Izquierdo A, Althawadi S, Araúz AB, Ben-Ami R, Bharat A, Calvo B, Desnos-Ollivier M, Escandón P, Gardam D, Gunturu R, Heath CH, Kurzai O, Martin R, Litvintseva AP, Cuomo CA. 2020. Tracing the evolutionary history and global expansion of Candida auris using population genomic analyses. mBio 11:e03364-19. doi:10.1128/mBio.03364-19 32345637 PMC7188998

[10] Centers for Disease Control and Prevention. 2024. Antifungal susceptibility testing and interpretation. Available from: https://www.cdc.gov/candida-auris/hcp/laboratories/antifungal-susceptibility-testing.html. Retrieved 10 Mar 2025.

[11] Rybak JM, Barker KS, Muñoz JF, Parker JE, Ahmad S, Mokaddas E, Abdullah A, Elhagracy RS, Kelly SL, Cuomo CA, Rogers PD. 2022. In vivo emergence of high-level resistance during treatment reveals the first identified mechanism of amphotericin B resistance in Candida auris. Clin Microbiol Infect 28:838–843. doi:10.1016/j.cmi.2021.11.024 34915074 PMC9467277

[12] Sharma C, Kumar N, Pandey R, Meis JF, Chowdhary A. 2016. Whole genome sequencing of emerging multidrug resistant Candida auris isolates in India demonstrates low genetic variation. New Microbes New Infect 13:77–82. doi:10.1016/j.nmni.2016.07.003 27617098 PMC5006800

[13] Rhodes J, Fisher MC. 2019. Global epidemiology of emerging Candida auris. Curr Opin Microbiol 52:84–89. doi:10.1016/j.mib.2019.05.008 31279224

[14] Escandón P, Chow NA, Caceres DH, Gade L, Berkow EL, Armstrong P, Rivera S, Misas E, Duarte C, Moulton-Meissner H, Welsh RM, Parra C, Pescador LA, Villalobos N, Salcedo S, Berrio I, Varón C, Espinosa-Bode A, Lockhart SR, Jackson BR, Litvintseva AP, Beltran M, Chiller TM. 2019. Molecular epidemiology of Candida auris in Colombia reveals a highly related, countrywide colonization with regional patterns in amphotericin B resistance. Clin Infect Dis 68:15–21. doi:10.1093/cid/ciy411 29788045

[15] Ceballos-Garzon A, Peñuela A, Valderrama-Beltrán S, Vargas-Casanova Y, Ariza B, Parra-Giraldo CM. 2023. Emergence and circulation of azole-resistant C. albicans, C. auris and C. parapsilosis bloodstream isolates carrying Y132F, K143R or T220L Erg11p substitutions in Colombia. Front Cell Infect Microbiol 13:1136217. doi:10.3389/fcimb.2023.1136217 37026059 PMC10070958

[16] Misas E, Escandón P, McEwen JG, Clay OK. 2019. The LUFS domain, its transcriptional regulator proteins, and drug resistance in the fungal pathogen Candida auris. Protein Sci 28:2024–2029. doi:10.1002/pro.3727 31503375 PMC6798126

[17] CLSI. 2017. Reference method for broth dilution antifungal susceptibility testing of yeasts. In CLSI Standard M27, 4th ed. Clinical and Laboratory Standards Institute, Wayne, PA.

[18] CDC. 2021. CDC antibiotic resistance laboratory network: guidance for whole genome sequencing and analysis of Candida auris. ARLABnetwork.

[19] Suphavilai C, Ko KKK, Lim KM, Tan MG, Boonsimma P, Chu JJK, Goh SS, Rajandran P, Lee LC, Tan KY, et al.. 2024. Detection and characterisation of a sixth Candida auris clade in Singapore: a genomic and phenotypic study. Lancet Microbe 5:100878. doi:10.1016/S2666-5247(24)00101-0 39008997

[20] Argimón S, Abudahab K, Goater RJE, Fedosejev A, Bhai J, Glasner C, Feil EJ, Holden MTG, Yeats CA, Grundmann H, Spratt BG, Aanensen DM. 2016. Microreact: visualizing and sharing data for genomic epidemiology and phylogeography. Microb Genom 2:e000093. doi:10.1099/mgen.0.000093 28348833 PMC5320705

[21] Noguera MC, Orozco S, Lizarazo D, Escandón P. 2024. Prevalencia de Candida auris en departamentos de Colombia durante la vigilancia por el laboratorio (2018-2021). Infect 28:241–245. doi:10.22354/24223794.1202

[22] Armstrong PA, Rivera SM, Escandon P, Caceres DH, Chow N, Stuckey MJ, Díaz J, Gomez A, Vélez N, Espinosa-Bode A, Salcedo S, Marin A, Berrio I, Varón C, Guzman A, Pérez-Franco JE, Escobar JD, Villalobos N, Correa JM, Litvintseva AP, Lockhart SR, Fagan R, Chiller TM, Jackson B, Pacheco O. 2019. Hospital-associated multicenter outbreak of emerging fungus Candida auris, Colombia, 2016. Emerg Infect Dis 25:1339–1346. doi:10.3201/eid2507.180491 31211679 PMC6590770

[23] Berrio I, Caceres DH, Coronell R W, Salcedo S, Mora L, Marin A, Varón C, Lockhart SR, Escandón P, Berkow EL, Rivera S, Chiller T, Vallabhaneni S. 2021. Bloodstream infections with Candida auris among children in Colombia: clinical characteristics and outcomes of 34 cases . J Pediatric Infect Dis Soc 10:151–154. doi:10.1093/jpids/piaa038 32373928

[24] Caceres DH, Rivera SM, Armstrong PA, Escandon P, Chow NA, Ovalle MV, Díaz J, Derado G, Salcedo S, Berrio I, Espinosa-Bode A, Varón C, Stuckey MJ, Mariño A, Villalobos N, Lockhart SR, Chiller TM, Prieto FE, Jackson BR. 2020. Case–case comparison of Candida auris versus other Candida species bloodstream infections: results of an outbreak Investigation in Colombia. Mycopathologia 185:917–923. doi:10.1007/s11046-020-00478-1 32860564

[25] Morales-López SE, Parra-Giraldo CM, Ceballos-Garzón A, Martínez HP, Rodríguez GJ, Álvarez-Moreno CA, Rodríguez JY. 2017. Invasive infections with multidrug-resistant yeast Candida auris, Colombia. Emerg Infect Dis 23:162–164. doi:10.3201/eid2301.161497 27983941 PMC5176232

[26] Cortés JA, Ruiz JF, Melgarejo-Moreno LN, Lemos EV. 2020. Candidemia in Colombia. Biomedica 40:195–207. doi:10.7705/biomedica.4400 32220174 PMC7357379

[27] Cortés JA, Reyes P, Gómez CH, Cuervo SI, Rivas P, Casas CA, Sánchez R. 2014. Clinical and epidemiological characteristics and risk factors for mortality in patients with candidemia in hospitals from Bogotá, Colombia. Braz J Infect Dis 18:631–637. doi:10.1016/j.bjid.2014.06.009 25181401 PMC9425269

[28] Rodríguez AZ, Gómez C de B, Restrepo CAA, Parra HH, Arteaga MA, Moreno AR, Marín AG. 2010. Susceptibility to fluconazole and voriconazole of Candida species isolated from intensive care units patients in Medellin, Colombia (2001-2007). Rev Iberoam Micol 27:125–129. doi:10.1016/j.riam.2010.04.001 20450982

[29] Ortiz-Roa C, Valderrama-Rios MC, Sierra-Umaña SF, Rodríguez JY, Muñetón-López GA, Solórzano-Ramos CA, Escandón P, Alvarez-Moreno CA, Cortés JA. 2023. Mortality caused by Candida auris bloodstream infections in comparison with other Candida species, a multicentre retrospective cohort. J Fungi (Basel) 9:715. doi:10.3390/jof9070715 37504704 PMC10381160

[30] Nucci M, Queiroz-Telles F, Alvarado-Matute T, Tiraboschi IN, Cortes J, Zurita J, Guzman-Blanco M, Santolaya ME, Thompson L, Sifuentes-Osornio J, Echevarria JI, Colombo AL, on behalf of the Latin American Invasive Mycosis Network. 2013. Epidemiology of candidemia in Latin America: a laboratory-based survey. PLoS One 8:e59373. doi:10.1371/journal.pone.0059373 23527176 PMC3601956

[31] Hirayama T, Miyazaki T, Sumiyoshi M, Ito Y, Ashizawa N, Takeda K, Iwanaga N, Takazono T, Yamamoto K, Izumikawa K, Yanagihara K, Makimura K, Tsukamoto K, Kohno S, Mukae H. 2023. Echinocandin resistance in Candida auris occurs in the murine gastrointestinal tract due to FKS1 mutations. Antimicrob Agents Chemother 67:e0124322. doi:10.1128/aac.01243-22 36920237 PMC10112215

[32] Price TK, Mirasol R, Ward KW, Dayo AJ, Hilt EE, Chandrasekaran S, Garner OB, de St Maurice A, Yang S. 2021. Genomic characterizations of Clade III lineage of Candida auris, California, USA. Emerg Infect Dis 27:1223–1227. doi:10.3201/eid2704.204361 33755003 PMC8007294

[33] Misas E, Escandón PL, Gade L, Caceres DH, Hurst S, Le N, Min B, Lyman M, Duarte C, Chow NA. 2024. Genomic epidemiology and antifungal-resistant characterization of Candida auris, Colombia, 2016-2021. mSphere 9:e0057723. doi:10.1128/msphere.00577-23 38299868 PMC10900874

[34] Li J, Coste AT, Liechti M, Bachmann D, Sanglard D, Lamoth F. 2023. Novel ERG11 and TAC1b mutations associated with azole resistance in Candida auris. Antimicrob Agents Chemother 65:e02663-20. doi:10.1128/aac.02663-20 33619054 PMC8092887

[35] Czajka KM, Venkataraman K, Brabant-Kirwan D, Santi SA, Verschoor C, Appanna VD, Singh R, Saunders DP, Tharmalingam S. 2023. Molecular mechanisms associated with antifungal resistance in pathogenic Candida species. Cells 12:2655. doi:10.3390/cells12222655 37998390 PMC10670235

[36] Eliaš D, Tóth Hervay N, Gbelská Y. 2024. Ergosterol biosynthesis and regulation impact the antifungal resistance and virulence of Candida spp. Stresses 4:641–662. doi:10.3390/stresses4040041

[37] Dufourc EJ. 2008. Sterols and membrane dynamics. J Chem Biol 1:63–77. doi:10.1007/s12154-008-0010-6 19568799 PMC2698314

[38] Izumi H, Nafie LA, Dukor RK. 2024. Effect of conformational variability on the drug resistance of Candida auris ERG11p and FKS1. ACS Omega 9:19816–19823. doi:10.1021/acsomega.3c08134 38737078 PMC11080008

